# Traditional values and adolescent depression: Unraveling mediating mechanisms through self-esteem

**DOI:** 10.1371/journal.pone.0331660

**Published:** 2025-09-15

**Authors:** Yayu Jia, Chen Zhang

**Affiliations:** 1 Department of Anthropology, School of Social Research, Renmin University of China, Beijing, China; 2 Department of Social Work, School of Law, Minnan Normal University, Zhangzhou, China; Jawaharlal Institute of Postgraduate Medical Education and Research, INDIA

## Abstract

Drawing on nationally representative data from the 2018 China Family Panel Studies (CFPS), this research examines the mechanisms through which traditional values influence adolescent depressive symptoms via self-esteem. Utilizing Karlson-Holm-Breen (KHB) decomposition analysis with a sample of 4,217 adolescents aged 10−19 years, we found that overall traditional values demonstrated a nonsignificant total effect (β = −0.471, p = 0.116) yet exhibited a significant indirect effect through self-esteem (β = −0.447, p < 0.01 = 0.001, 95% CI [−0.716, −0.177]). Material-oriented and mortality-related values, including pursuit of wealth (β = 0.222, p = 0.178), avoiding social disapproval (β = −0.388, p = 0.031), and posthumous remembrance (β = −0.065, p = 0.648), demonstrated no significant mediation effects. Future-oriented traditional values manifested complete mediation through self-esteem, with significant indirect effects for intimate relations (β = −0.155, p = 0.026), achievement orientation (β = −0.226, p = 0.005), family cohesion (β = −0.255, p = 0.019), lineage continuation (β = −0.159, p = 0.020), and offspring success (β = −0.166, p = 0.042). Hedonic value orientation manifested partial mediation with both significant direct (β = −0.410, p = 0.050) and indirect effects (β = −0.312, p = 0.001). These findings illuminate how traditional values influence adolescent mental health in contemporary China, where distal life-course values operate through self-evaluative mechanisms, while proximate experiential values maintain direct psychological effects.

## Introduction

The prevalence of depressive symptoms in adolescence has seen a concerning rise worldwide, becoming a pressing public health crisis. Epidemiological studies estimate 10–20% of adolescents now experience significant depressive symptoms, with rates increasing over the past decade [1]. Depressive symptoms in adolescence encompass persistent negative moods, loss of interest and pleasure, fatigue, sleep and appetite changes, and other cognitive-affective disturbances, although not necessarily amounting to clinical diagnosis of depression [2]. Nonetheless, elevated depressive symptoms critically impact adolescents’ quality of life and functioning [3]. This troubling situation underscores the urgent need to identify protective factors that can strengthen resilience and alleviate depressive tendencies in adolescents. Low self-esteem is a risk factor for depression, with longitudinal studies showing it prospectively predicts increases in depressive symptoms from adolescence to adulthood [4,5]. As adolescence is a critical period for developing self-esteem when adolescents are forming self-identity, interventions boosting self-esteem may protect against escalating depressive symptoms.

The social identity theory proposes that individuals tend to conform to the values and norms of their group in order to maintain a positive social identity and high self-esteem [6]. Adherence to culturally-ingrained traditional values, in particular, may enhance self-esteem by providing a sense of belonging, acceptance, and purpose. While traditional values may not always align with mainstream or majority views in times of rapid social change, they represent a powerful source of cultural identity. Traditional values reflect shared ideas about what behaviors and ideals are desirable to uphold, which have been emphasized and propagated over generations. Specifically, within the Chinese context, traditional values are heavily influenced by a Confucian heritage that emphasizes collectivism and familism over individualism [7]. These values prioritize group harmony, filial piety, and familial prosperity. This study operationalizes these values by examining the perceived importance of specific life goals that reflect this tradition, such as achieving career success (achievement), securing the family’s future through the pursuit of wealth, maintaining strong family bonds (family harmony, intimate relations), ensuring the continuation of the family name (lineage continuation), and guaranteeing the success of the next generation (successful children) [8]. These stand in contrast to more individualistic values that have become prominent through globalization. Nonetheless, it remains unclear how endorsing these traditional values might impact depressive symptoms. Thus, a crucial question arises: does endorsing traditional life values indirectly influence levels of depression through its effect on self-esteem?

Adolescents born in the new millennium have grown up in an environment characterized by unprecedented access to information and diverse cultural influences, primarily facilitated by the internet and social media [9]. This exposure has led to a greater awareness of alternative lifestyles, beliefs, and value systems from around the world. As a result, many of them may find themselves in a state of cultural pluralism, where traditional values handed down by previous generations are challenged by a multitude of new ideas and perspectives. This clash between traditional values and the diversity of thought in the new millennium can create a sense of dissonance for today’s adolescents [10]. On one hand, they may feel the weight of traditional expectations and norms, which may include values related to family, religion, and social conformity. On the other hand, they are exposed to a globalized world that celebrates individualism, diversity, and personal choice [9]. Consequently, some adolescents may experience a sense of cultural dislocation, struggling to reconcile the values of their cultural heritage with the dynamic and ever-changing world they inhabit [11]. They may question the relevance of traditional values in the face of contemporary challenges and opportunities. This tension can lead to feelings of identity crisis, as they grapple with defining their own set of values and beliefs in an increasingly pluralistic society [12,13]. In this context, it is important to recognize that the impact of traditional values on adolescents in the new millennium is not uniform. Some may choose to embrace and uphold traditional values, finding meaning and stability in them. Others may seek a balance between tradition and modernity, while still others may reject traditional values altogether in favor of a more individualistic or secular outlook.

Connections between traditional values endorsement, self-esteem, and adolescent depression symptoms remain poorly understood. Examining these potential linkages can inform efforts to combat rising adolescent depression globally. The present study aims to address this research gap by investigating the relationships between endorsement of traditional life values, self-esteem, and depressive symptoms in Chinese adolescents. Findings will provide novel evidence on the culturally traditional benefits that upholding intimate relationships, family bonds, achievement, and community may confer for adolescent mental health.

## Literature review

### Self-esteem and depressive symptoms

Numerous studies have demonstrated a link between low self-esteem and depression. According to the vulnerability model, low self-esteem is an established risk factor that leads individuals to be more vulnerable to developing depression [14]. This view is supported by substantial empirical evidence using longitudinal designs.

In a prospective study following individuals from adolescence through adulthood, Orth and Robins found that low self-esteem predicted subsequent increases in depressive symptoms. The association was consistent across gender, age groups, and national samples [15]. Another longitudinal study by Steiger et al. reported that decreases in self-esteem during adolescence predicted depressive symptoms in adulthood two decades later [5].

Further evidence comes from longitudinal studies employing cross-lagged panel analyses to ascertain causal predominance between self-esteem and depression. For instance, Sowislo and Orth analyzed data across three longitudinal studies, confirming that self-esteem prospectively predicted depression, but depression did not predict later self-esteem [16]. Similar results were obtained in studies with early adolescent samples [17].

Taken together, extensive empirical research robustly supports the vulnerability model that low self-esteem functions as an antecedent risk factor for depression onset and worsening symptoms over time. This effect persists from adolescence through adulthood, highlighting the enduring impacts of low self-esteem on mental health. Enhancing self-esteem, especially during formative developmental stages, may confer resilience against depression. Indeed, a meta-analysis of school-based prevention programs found that interventions successful in boosting self-esteem also led to a reduction in depressive symptoms, supporting its role as a protective factor [18]. Adolescence marks a critical period for developing self-esteem among adolescents, when they are actively forming self-identity and seeking social approval. Interventions that boost adolescents’ self-esteem may confer protection against escalating depressive symptoms over vulnerable developmental periods.

### Traditional life values and depressive symptoms

Traditional values are long-standing in human society and emphasize national pride, respect for authority, obedience, parent-child ties, and marriage [19]. These traditional values often serve as the moral compass and social fabric within societies, shaping individuals’ perceptions and behaviors. When considering the relationship between traditional values and their impact on well-being, several possibilities emerge, shedding light on the complexity of this association.

One possibility is that person-environment value congruence benefits well-being. Sagiv and Schwartz highlighted the importance of person-environment value congruence in influencing well-being [20]. When personal values matched prevailing values in one’s environment, higher subjective well-being was reported. This demonstrates that the impacts of values on well-being depend on the extent to which the environment supports or inhibits their pursuit. Many studies have found that acculturation can significantly affect depressive symptoms [21].

Another possibility is that person-environment value congruence does not necessarily benefit well-being. As Waldeck et al. pointed out, adaptability and psychological flexibility are distinct yet overlapping constructs that both contribute to mental health [22]. This suggests that individuals with high adaptability can maintain psychological well-being even when their values partially diverge from their surroundings. Firstly, individual adaptability and resilience also impact mental health. With self-regulation skills, one can achieve decent psychological states even when values do not fully align with the environment. Doorley et al. further argued that psychological flexibility enables individuals to pursue personally meaningful goals even in the presence of conflicting or challenging contexts [23]. Such environments, by necessitating adaptive responses, can actually strengthen one’s capacity to grow and thrive. Moderately inconsistent values can motivate people to actively adapt, broadening horizons, which can also be positive.

Secondly, total conformity in values may lead to fixed mindsets, hindering personal growth. Moreover, achieving complete consistency between values and environment is unrealistic in life. What matters is maintaining openness, tolerance and mutual understanding when differences emerge – hallmarks of psychological wellness. Additionally, personal values evolve over time. Excessively consistent values may prevent adapting to future changes.

A final possibility is that impacts on well-being depend on the type of traditional values. Kasser and Ryan showed that financial success values, compared to intimacy and growth values, associated with more depressive symptoms over time among young adults [24]. Here, “financial success values” are considered extrinsic, as they are contingent on external approval and rewards, whereas “intimacy and growth values” are intrinsic, being inherently satisfying and aligned with core psychological needs. Researchers explained that values like materialism may undermine psychological need satisfaction and cultivate social comparisons. Among Latino adolescents, greater endorsement of family cultural values like familism and filial obligations associated with lower depressive symptoms, especially for females [25]. Also, larger cultural value gaps regarding affiliative obedience between Mexican American parents and adolescents related to greater adolescent depressive symptoms [26]. Moreover, different dimensions of familism values had differential impacts on depressive symptoms in Mexican-origin adolescents [27]. Chinese research has also demonstrated the salience of traditional cultural values in adolescent psychological adjustment, with filial piety showing both protective and risk effects depending on its specific dimensions [28].

### Traditional life values and self-esteem

Self-esteem is an individual’s evaluation of the extent to which he or she practices his or her cultural value standards [29]. The relationship between traditional life values and self-esteem has been explored from multiple theoretical perspectives.

The cultural norm-fulfillment perspective posits that self-esteem results from adhering to the values and norms of one’s culture [30]. Through socialization, individuals internalize the values and prescribed roles of their culture. This process aligns with sociologist George Herbert Mead’s symbolic interactionism theory, highlighting that self-concept is significantly shaped by social interactions and cultural symbols [31]. Fulfilling these culturally-defined roles and actualizing the cultural values is posited to lead to feelings of self-worth and high self-esteem [32].

The interpersonal-belonging perspective regards self-esteem as an internal gauge of the quality of one’s social bonds and inclusionary status [33]. The need to belong and feel socially connected is argued to be a fundamental human motivation. Self-esteem is proposed to function as a sociometer that monitors the degree to which an individual is being accepted or rejected by other people. The pursuit of traditional life values often leads to positive feedback and validation from others. As individuals strive to achieve these values, they may receive praise, admiration, and recognition for their efforts and accomplishments. Such external affirmation serves as a powerful source of self-esteem enhancement.

Empirical evidence reveals complex links between values and self-esteem. Self-enhancement values like achievement and power relate positively to self-esteem, while self-transcendence values like benevolence relate negatively [34].

Research specifically looking at Israeli adolescents has found their self-esteem is higher when they hold similar self-enhancement and self-transcendence values as their classmates [35]. First generation immigrant adolescents show a particularly strong negative relationship between value differentiation across contexts and self-esteem, suggesting value consistency may be especially important for their self-concept [36]. Among Turkish adolescents, those with higher personal values showed higher self-esteem and meaning in life [37]. Synthesizing these varied findings suggests a complex pattern where the relationship between values and self-esteem is moderated by the cultural context and the social environment. These seemingly contradictory findings illustrate that value impact on self-evaluation depends not only on content but also on alignment with the immediate social world. While self-transcendence negatively related to self-esteem in one study, it showed positive relations when congruent with peers in another, demonstrating the importance of social context. In individualistic societies, self-enhancement may be a more direct route to self-esteem, whereas in collectivistic societies, fulfilling communal duties may be equally or more important for self-worth.

### Theoretical Framework

This study is guided by a conceptual framework that integrates three core theoretical perspectives to explain the pathway from traditional values to depressive symptoms. First, the Vulnerability Model of Depression posits that low self-esteem is a significant risk factor that predisposes individuals to depression [4,14]. Second, the Cultural Norm-Fulfillment Perspective and Sociometer Theory explain how self-esteem is formed. The former suggests that self-esteem is derived from successfully adhering to the values and standards of one‘s culture [30], while the latter proposes that self-esteem functions as an internal gauge of social acceptance and belonging [33].

Together, these theories support a mediational model where cultural values influence mental health through self-evaluation. Our conceptual framework posits a three-component pathway wherein endorsement of traditional values functions as the predictor variable, self-esteem operates as the mediating mechanism, and depressive symptoms serve as the outcome variable. We hypothesize that adolescents internalize traditional values as standards for self-worth evaluation. The process of fulfilling or endorsing these values enhances both social acceptance and self-approval, leading to increased self-esteem in the first stage of mediation. Subsequently, this enhanced self-esteem acts as a protective psychological resource that buffers against the development of depressive symptoms in the second stage. This theoretical framework provides compelling rationale for positioning self-esteem as the central mediating variable in our analytical model.

### The current study

The aim of the current study is to examine the relationships between endorsement of traditional life values, self-esteem, and depressive symptoms among adolescents.

Based on previous research, endorsement of traditional values prevalent in one’s cultural context can promote adolescents’ self-esteem, which in turn reduces depressive symptoms. However, due to ongoing societal changes, some traditional values may be becoming less important or endorsed currently. The mechanisms relating values and mental health may differ across values.

Therefore, we propose the following hypotheses:

H1: Self-esteem mediates the relationship between overall endorsement of traditional life values and adolescent depressive symptoms. Specifically, higher endorsement of traditional values will predict higher self-esteem (path a), and higher self-esteem will predict lower depressive symptoms (path b).
H2: The mediating role of self-esteem will differ based on the value’s temporal and experiential proximity to adolescent life. (a) For distal, life-course oriented values (e.g., achievement, family continuation), endorsement will be fully mediated by self-esteem, as these values primarily shape self-concept without having a direct, tangible impact on daily mood. (b) For proximal, experiential values (e.g., pleasure), endorsement will be partially mediated, exerting both a direct effect on depressive symptoms (through immediate experience) and an indirect effect through self-esteem.

**Fig 1:**
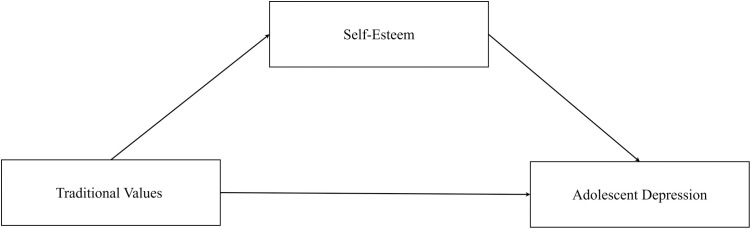
The conceptual framework.

This study aims to test these hypotheses by examining the associations between endorsement of prevailing traditional values, self-esteem, and depression levels among adolescents. Findings can provide insight into the nuanced relationships between sociocultural values, self-views, and adolescent psychological well-being amidst generational shifts in value orientations. The conceptual framework is illustrated in Fig 1.

## Methods

### Data

The data for this study comes from the 2018 wave of the China Family Panel Studies (CFPS). The CFPS is a nationally representative, annual longitudinal survey of Chinese communities, families, and individuals. The CFPS project received ethical approval from the Peking University Biomedical Ethics Committee (approval number: IRB00001052–14010) with informed consent obtained from all participants (and parental consent for minors), and no additional ethical approval was required for this secondary data analysis. Data were accessed on 01/09/2022.

The total CFPS 2018 sample consists of 37,354 individuals. According to the WHO definition of adolescents as persons aged 10–19 years [38], we retained adolescent respondents between 10–19 years old, giving a subsample of 4,217 adolescents.

### Measurement

RSES is used to assess the self-esteem of adolescents. The scale is one of the most commonly used self-esteem measurement tools in psychology [39]. It consists of 5 forward-scoring and 5 reverse-scoring questions, with responses ranging from 1 to It is made on a scale of 5 (strongly disagree to strongly agree), with a total score between 10 and 50. The higher the final score, the higher the level of self-esteem. The Cronbach’s alpha for this measure was 0.644 in this study. While this value is below the commonly cited 0.70 threshold, it is often considered acceptable for scales with a small number of items, and similar alpha levels have been reported in large, diverse survey samples [40].

CESD-8 has a total of 8 items (including 2 reverse-scored items). Each item is divided into “almost never (less than a day)”, “sometimes (1 to 2 days)”, “often (3 to 4 days)”, and “Most of the time (5 to 7 days)” are four options, each assigned a score of 0–3, with a total score ranging from 0 to 24. The higher the score, the more serious the respondent’s tendency to depression in the past week. The Cronbach’s alpha for this measure was 0.721 in this study.

Participants’ endorsement of traditional values was measured using a 10-item scale assessing pursuit of wealth, avoiding being disliked, pleasure, Intimate relations, avoiding loneliness, achievement, being missed after death, family harmony, continuing the family line, and successful children. The items were selected from the adult questionnaire of the China Family Panel Studies (CFPS), designed to measure respondents’ core life attitudes and values. While the CFPS is a validated and widely used longitudinal study, the construction of these specific items into a single “traditional values” scale is an approach developed for this study to capture the multifaceted nature of traditionalism pertinent to Chinese society. The scale demonstrated acceptable internal consistency in our sample (α = 0.782). Participants rated each value from 1 = “Not at all important” to 5 = “Extremely important”. Scores were averaged to create an overall traditional values endorsement score. Higher scores indicated stronger adherence to traditionalism.

### Data analysis

To test our hypotheses, we conducted a mediation analysis to decompose the total effect of traditional values on depressive symptoms into a direct effect and an indirect effect operating through self-esteem. We employed the Karlson-Holm-Breen (KHB) mediation analysis method to examine and quantify these effects.

The KHB method, developed by Karlson et al., is a relatively recent advancement in mediation analysis [41]. It offers an extension to traditional mediation analysis by addressing the issue of mediator-outcome confounding, which can lead to biased estimates of mediation effects. KHB analysis provides adjusted estimates that consider the influence of covariates on both the mediator and the outcome, thus enhancing the accuracy of mediation assessment.

The primary principle underlying KHB mediation analysis is to rectify potential bias arising from the presence of uncontrolled confounders in the mediator-outcome relationship. This bias can distort the estimation of the indirect (mediation) effect. KHB accomplishes this by introducing an additional adjustment to the traditional mediation analysis model.

The data analysis for this study was conducted using Stata/MP 17.0.

## Results

Table 1 presents sample characteristics of the adolescents in the study. The sample exhibited a mean self-esteem score of 38.79 (SD = 4.56), indicating a moderate level of self-esteem. Their average value score was 3.89 (SD = 0.57), reflecting the extent to which they endorsed traditional values. In terms of mental well-being, the adolescents reported an average of 4.27 (SD = 3.22) on the scale measuring depressive symptoms. Age-wise, the sample had a mean age of 14.38 years (SD = 2.91). Gender distribution revealed that 52.96% of the participants were male. Moreover, the majority of the adolescents (86.84%) were currently attending school, while 13.16% were not in school. Approximately 30.13% of the participants were affiliated with the Communist Youth League. Furthermore, mobile phone usage was prevalent among the adolescents, with 64.99% of them reporting regular mobile phone usage.

**Table 1:** Characteristics of Teenagers.

	M(SD)	%
Self-esteem	38.79(4.56)	
Value	3.89(0.57)	
Depressive symptoms	4.27(3.22)	
Age	14.38(2.91)	
Gender (Male)		52.96
School status		
Not in school		13.16
In school		86.84
Communist Youth League Member		30.13
Using a mobile phone		64.99

In this study, we conducted a mediation analysis using the KHB method to investigate the relationships among traditional values (both overall and specific values), self-esteem, and depressive symptoms in adolescents aged 10–19 years. KHB analysis results are presented in Table 2.

**Table 2:** Mediation analysis results.

Independent variable	Mediating variable	Dependent variable: depressive symptoms		
		Total effect	Direct effect	Indirect effect
Traditional value (overall)	Self-esteem	−0.471(0.299) [-1.057,0.116] p = 0.116	−0.024(0.306) [-0.623,0.575] p = 0.937	−0.447**(0.137) [-0.716,-0.177] p = 0.001
wealth	Self-esteem	0.222(0.165) [-.101,0.544] p = 0.178	0.122(0.165) [-0.202,0.445] p = 0.462	0.100(0.071) [-0.038,0.239] p = 0.157
Avoiding being disliked	Self-esteem	−0.388*(0.180) [-0.740,-0.036] p = 0.031	−.024(0.181) [-0.597,0.111] p = 0.178	−0.145(0.077) [-0.297,0.007] p = 0.061
Pleasure	Self-esteem	−0.723***(0.204) [-1.123,-0.322] p = 0.000	−.410*(0.209) [-0.820,-0.001] p = 0.050	−0.312**(0.092) [-0.493,-0.131] p = 0.001
Intimate relations	Self-esteem	0.009(0.155) [0.296,0.313] p = 0.954	0.164(0.157) [-0.143,0.471] p = 0.296	−0.155*(0.069) [-0.291,-0.019] p = 0.026
Avoiding loneliness	Self-esteem	−0.424**(0.161) [-0.740,0.108] p = 0.009	−0.245(0.163) [-0.565,0.075] p = 0.133	−0.179*(0.071) [-0.317,-0.041] p = 0.011
Achievement	Self-esteem	−0.193(0.175) [-0.535,0.149] p = 0.269	0.033(0.177) [-0.314,0.381] p = 0.852	−0.226**(0.081) [-0.382,-0.070] p = 0.005
Being missed after death	Self-esteem	−0.065(0.142) [-0.342,0.212] p = 0.648	0.042(0.142) [-0.236,0.321] p = 0.765	−0.107(0.061) [-0.227,0.013] p = 0.079
Family harmony	Self-esteem	−0.250(0.245) [-.730,0.230] p = 0.308	0.006(0.247) [-0.479,0.490] p = 0.982	−0.255*(0.109) [-0.469,-0.042] p = 0.019
Continuing the family line	Self-esteem	0.014(0.151) [-0.282,0.310] p = 0.927	0.173(0.153) [-0.126,0.472] p = 0.257	−0.159*(0.069) [-0.294,-0.025] p = 0.020
Successful children	Self-esteem	−0.108(0.187) [-0.475,0.258] p = 0.562	0.057(0.188) [-0.312,0.426] p = 0.761	−0.166*(0.082) [-0.325,-0.006] p = 0.042

*p < 0.05. **p < 0.01. ***p < 0.001.

The overall traditional value exhibited a total effect of −0.471 (p = 0.116) on depressive symptoms. Surprisingly, the direct effect of traditional value on depressive symptoms was non-significant at −0.024 (p = 0.937). However, a significant indirect effect through self-esteem was observed at −0.447 (p = 0.001), with a confidence interval of [−0.716, −0.177]. This suggests that traditional values have an impact on depressive symptoms, primarily mediated by self-esteem. The suppressive effect of self-esteem highlights the complexity of the relationship between traditional values and depressive symptoms.

For pursuit of wealth values, the total effect of pursuit of wealth on depressive symptoms was 0.222 (β = 0.222, p = 0.178), with a non-significant direct effect of 0.122 (β = 0.122, p = 0.462) and a non-significant indirect effect through self-esteem of 0.100 (β = 0.100, p = 0.157). This indicates no significant mediation effect of self-esteem.

Similarly, the total effect of avoiding being disliked on depressive symptoms was −0.388 (β = −0.388, p = 0.031). The direct effect was non-significant at −0.024 (β = −0.024, p = 0.178), and the indirect effect through self-esteem was −0.145 (β = −0.145, p = 0.061), again indicating no significant mediation effect.

For being missed after death, the total (β = −0.065, p = 0.648), direct (β = 0.042, p = 0.765), and indirect effects via self-esteem (β = −0.107, p = 0.079) were all non-significant, demonstrating no mediation effect.

Intimate relations had a total effect of 0.009 (p = 0.954) on depressive symptoms. The non-significant direct effect of 0.164 (p = 0.296) suggests that the relationship between intimate relations and depressive symptoms is influenced by other variables not included in this analysis. However, the indirect effect through self-esteem of −0.155 (p = 0.026) signifies that self-esteem plays a suppressive role, partially mediating the relationship between intimate relations and depressive symptoms.

The total effect of achievement on depressive symptoms was −0.193 (p = 0.269). Despite the non-significant direct effect of 0.033 (p = 0.852), the presence of suppression effects becomes evident. The indirect effect through self-esteem, which is −0.226 (p = 0.005), indicates that self-esteem suppresses the relationship between achievement and depressive symptoms, revealing a more complex interplay.

Family harmony exhibited a total effect of −0.250 (p = 0.308) on depressive symptoms. Similarly, the non-significant direct effect of 0.006 (p = 0.982) suggests that other factors may be at play. The indirect effect through self-esteem, however, is −0.255 (p = 0.019), signifying the presence of suppression effects. Self-esteem suppresses the relationship between family harmony and depressive symptoms, emphasizing the role of self-esteem in this context.

Continuing the family line had a total effect of 0.014 (p = 0.927) on depressive symptoms. The non-significant direct effect of 0.173 (p = 0.257) implies the involvement of other variables. The indirect effect through self-esteem, which is −0.159 (p = 0.020), highlights the suppressive nature of self-esteem in mediating the relationship between continuing the family line and depressive symptoms.

Successful children exhibited a total effect of −0.108 (p = 0.562) on depressive symptoms. Despite the non-significant direct effect of 0.057 (p = 0.761), the indirect effect through self-esteem was −0.166 (p = 0.042). This suggests that self-esteem acts as a suppressor variable in the relationship between successful children and depressive symptoms, indicating a more intricate pathway in understanding this association.

In contrast to the above variables, avoiding loneliness exhibited no suppression effects. Avoiding loneliness had a total effect of −0.424 (p = 0.009) on depressive symptoms. The direct effect was −0.245 (p = 0.133), and the indirect effect through self-esteem was −0.179 (p = 0.011). This indicates complete mediation, where the total effect is significant but the direct effect is not, once the mediator is included.

For pleasure, the total effect on depressive symptoms was −0.723 (p = 0.000). The direct effect was significant at −0.410 (p = 0.050), and the indirect effect through self-esteem was also significant at −0.312 (p = 0.001).

## Discussion

Our study investigated the mechanisms linking endorsement of traditional values and depressive symptoms in Chinese adolescents. Our study delves into the mediation chain involving ten different values, self-esteem, and depressive symptoms, shedding light on the intricate pathways through which various traditional values impact the emotional well-being of adolescents, providing valuable insights into the experience of heightened depressive symptoms.

Our findings support H1, demonstrating that self-esteem fully mediates the relationship between overall traditional values and depressive symptoms. This mediation mechanism extends previous research, which has established the negative association between traditional values and depressive symptoms [28] and the negative association between self-esteem and depressive symptoms [4]. Our study advances this understanding by demonstrating that traditional values operate through self-esteem to influence depressive symptoms. However, this overall pattern masks substantial heterogeneity across specific values, supporting H2 that different traditional values operate through distinct mechanisms, consistent with research documenting differential effects of specific cultural value dimensions.

For pursuit of wealth, avoiding being disliked, and being remembered after death, no mediation effect was found. Contemporary teens have limited direct engagement with finances, so valuing pursuit of wealth may not impact their self-concept or moods. As Gentina and Tang found, although material values are often present in adolescence, financial concepts like pursuit of wealth are not always internalized deeply enough to affect adolescents’ identity formation, particularly because they lack real financial independence or responsibility [42]. Avoiding being disliked showed no mediation, possibly because today’s adolescents with prevalent individualism care less about others’ opinions compared to previous collectivistic generations. Also, education equips adolescents to view social evaluations more objectively. Death remains a distant concept detached from teens’ everyday concerns. Therefore, these values did not affect depression indirectly through self-esteem.

In contrast, intimate relations, achievement, avoiding loneliness, family harmony, continuing the family line, and successful children showed full mediation. This finding strongly supports H2a. Contemporary teens lack direct exposure to these remote life events like career success, marital intimacy, or childrearing. Thus, endorsing these values could only influence depression by shaping the more immediate self-esteem. This aligns with the cultural norm-fulfillment perspective [30], where self-esteem is derived from embracing cultural ideals, even if those ideals are abstract or future-oriented. Without personally experiencing these life stages, adolescents do not have tangible appraisals to directly shape their moods. The full mediation effects shown for these values may also be explained by deeper ongoing sociocultural changes that make these traditional values less directly relevant and important to contemporary Chinese adolescents. Today’s adolescents, as digital-native Gen Z, have access to more diverse values including singleness, childfreeness, and work disengagement. This generation may view traditional values like achievement, familial piety, and continuity as less relevant and important.

For the avoidance of loneliness value, self-esteem also fully mediated the effect on lower depression. Actively fostering social bonds does not directly guarantee happiness or life satisfaction. Peer interactions can also be frustrating for adolescents. Hence, loneliness affects mental health indirectly by cultivating self-worth. This is consistent with sociometer theory [33], which posits that our sense of belongingness is a primary determinant of self-esteem. While avoidance of loneliness demonstrated full mediation like the other traditional values, it did not exhibit suppression effects. This highlights key differences between avoiding loneliness and values like achievement, family, and continuity. Firstly, avoiding loneliness emphasizes relational values that remain highly relevant to adolescents’ immediate priorities. Adolescents still care deeply about peer bonds despite generational change. In contrast, values like achievement and familial piety seem more disconnected from teens’ lived experiences. Secondly, avoiding loneliness is anchored more in psychological needs than localized social conventions. The desire for belonging is universal across cultures and generations. Comparatively, conformity to traditions like continuing the family line depends more on alignment with normative expectations.

Moreover, our exploration of values centered around pleasure unraveled a nuanced pattern with partial mediation effects, supporting H2b. Pleasure resonates with teens’ current life experiences, so their endorsement could directly reduce depressive affect. This direct effect aligns with research on intrinsic motivation, suggesting that pursuing inherently enjoyable activities directly enhances well-being, independent of self-evaluation [24]. Simultaneously, aligning with these values may foster positive self-views, indirectly decreasing depression. The relevance of these values may arise from contemporary adolescents’ greater resources, individualism, and self-focus, regardless of culture. Experiencing pleasure resonates with this generation’s priorities.

The intricate mechanisms linking sociocultural change, values, self-perceptions, and adolescent depressive symptoms revealed in this study hold important insights for enhancing adolescent mental health services, education, and policies.

Firstly, the findings highlight the need for a nuanced, tailored approach to promoting adolescent mental wellbeing – one that considers the impacts of both proximal and distal values. Traditions like achievement and familial piety, although eroded in relevance today, still shape adolescent self-esteems and thereby emotional states indirectly. Thus, interventions could acknowledge the subtle influences of these values in shaping adolescent self-concepts, while also recognizing their mismatch with adolescent priorities. Holistic strategies that help adolescents flexibly balance tradition and change may be beneficial.

Meanwhile, relational values like avoiding loneliness directly tap into adolescents’ core social motivations. Prioritizing belongingness and meaningful connections with others could provide an impactful avenue for lifting adolescent depressive affect directly. Peer counseling, mentorship programs, or social emotional learning emphasizing interpersonal skills may help buffer isolation and cultivate self-worth. Pleasure and enjoyment also aligned with adolescent priorities and directly reduced depressive symptoms. Providing adolescents opportunities for fun, recreation, and self-expression could also effectively enhance wellbeing.

Secondly, findings reveal the need to equip adolescents with self-regulatory skills and resilience to navigate value discrepancies. Maladaptive thought patterns like self-criticism, social comparison, or rumination could exacerbate distress when values clash with realities, as traditional norms frequently do. Cognitive-behavioral approaches building self-compassion, growth mindsets, and healthy coping may be invaluable. Simultaneously, improving social-emotional competencies like adaptability, empathy, and conflict management may help adolescent reconcile value differences with others.

Thirdly, schools should adopt inclusive, pluralistic value frameworks that resonate with diverse adolescents. With individualism ascendant, imposing rigid moral codes or traditional norms could alienate students ill-fitting predominant values. However, purposefully cultivating common values like mutual understanding despite differences could powerfully nurture belonging and self-worth. Schools could also provide platforms for open dialogue about evolving values between adolescents and elders, fostering intergenerational integration.

Fourthly, policies should address risk factors like discrimination that disproportionately affect adolescents feeling misaligned with traditional values. Lonely or rejected adolescents may be particularly vulnerable. Anti-bullying programs, crisis counseling, identity-affirming extracurricular groups, and safe reporting mechanisms could help support marginalized teens. Ensuring accessible mental health services in educational settings is also critically important.

Fifthly, media and public health campaigns should increase awareness of the diverse pathways linking values and wellbeing. Nuanced messaging could mitigate stigma around mental health help-seeking and normalize the inherent challenges of value conflicts. Reframing maladjustment as opportunity for growth may encourage resilience.

Finally, further research should investigate how adolescents in marginalized groups navigate value differences. LGBTQ+ individuals, ethnic minorities, or low-SES adolescents may face amplified value conflicts, isolation, and mental health risks. Understanding intersectional experiences could inform tailored interventions. Longitudinal studies could also enrich understanding of how sociocultural change reshapes values-mental health links across adolescence and the transition to adulthood. Our findings underscore the need to regularly re-examine long-held assumptions about values and wellbeing amidst generational shifts.

This study has several limitations. First, its cross-sectional design precludes causal inferences. Second, the Cronbach’s alpha for the RSES was modest, and future studies could benefit from using scales with higher internal consistency. Third, the “traditional values” scale was constructed from available items in the CFPS and, while internally consistent, has not been externally validated as a standalone scale. Finally, while we controlled for several demographic variables, other confounders could exist.

## Conclusion

Our study has unveiled the intricate sociocultural mechanisms that underlie the relationship between values and adolescent mental health. These findings beckon us to contemplate the evolving sociocultural landscape that contemporary adolescents find themselves in. As they are often characterized as the digital-native Generation Z, it’s apparent that they may have limited direct engagement with these traditional values. Our data serve as a poignant reflection of the changing tides in our society, where individualism and diverse value systems have come to the forefront, potentially rendering traditional values less relevant to the emotional well-being of today’s adolescents. Traditional values linked to distant life events primarily influence adolescent depression through the prism of self-esteem changes, serving as a mirror to the evolving sociocultural landscape. Conversely, values aligned with adolescents’ immediate experiences possess the inherent capacity to directly mold their emotional terrain, underscoring the significance of both remote and proximal values in comprehending and addressing the complex landscape of mental health among contemporary adolescents. These profound revelations hold broader implications for mental health interventions and educational strategies, compelling us to acknowledge and adapt to the evolving value systems of today’s adolescents to promote and safeguard their emotional well-being effectively.

In conclusion, this research provides profound insights into rapidly evolving sociocultural factors shaping adolescent mental health in the 21st century. By elucidating the mechanisms linking values, self-perceptions, and depressive symptoms, it compels deeper reflection on how to foster adolescent wellbeing through aligning interventions with shifting generational priorities. The findings underscore needs to holistically address values, skills, relationships, and risk factors through multi-faceted strategies involving schools, families, communities, media, and policy.
